# A Free-Market Environmentalist Enquiry on Spain’s Energy Transition along with Its Recent Increasing Electricity Prices

**DOI:** 10.3390/ijerph19159493

**Published:** 2022-08-02

**Authors:** William Hongsong Wang, Victor I. Espinosa, Jesús Huerta de Soto

**Affiliations:** 1 Instituto de Estudios Políticos y Relaciones Internacionales, Universidad Francisco Marroquín (Madrid Campus), Calle de Arturo Soria 245, 28033 Madrid, Spain; 2 La Fundación para el Avance de la Libertad, Calle del Marqués de la Ensenada 14, Primera Planta, Oficina 15, 28004 Madrid, Spain; 3 Public Policy Observatory, Universidad Autónoma de Chile, Santiago 7500912, Chile; 4 Nucleus of Humanities and Social Sciences, Universidad del Desarrollo, Av. La Plaza 700, Santiago 7610658, Chile; 5 Department of Applied Economics I, History and Economic Institutions and Moral Philosophy, Social and Legal Sciences Faculty, Rey Juan Carlos University, 28033 Madrid, Spain; huertadesoto@dimasoft.es

**Keywords:** free-market environmentalism, renewable energy, entrepreneurship, public policy, Spain, EU Green Deal, energy transition, electricity prices, decision making, public choice

## Abstract

This paper analyzes the Spanish energy transition’s general situation and its increasing electricity prices in recent years from a free-market environmentalist (FME) approach. We hypothesize and argue that high taxes, high government subsidies, and government industrial access restrictions breach private property rights, hindering Spain’s renewable energy (RE) development. Our paper discovers that Spain’s state-interventionist policies have increased the cost of the energy and power industries, leading to electricity prices remaining relatively high before and after the outbreak of the COVID-19 pandemic. After reviewing the literature on the FME approach and Spain’s case, a Box–Jenkins (ARIMA) model is used to clarify the economic performance of the Spanish electricity industry with a proposal for forecasting electricity prices. It is observed that Spain fails the EU and its national goal of providing an affordable energy price as a part of the green energy transition. Finally, free-market environmental solutions and policy reforms are proposed to facilitate Spain’s energy transition.

## 1. Introduction

The increasing electricity prices have recently caused great controversy in Spain [1]. Compared with other International Energy Agency (IEA) countries, even before the outbreak of the pandemic, Spain had the eighth highest electricity prices for industrial uses (122.7 USD/MWh) and the fifth highest prices (287.7 USD/MWh) for household consumers [2]. As of March 2022, the average monthly electricity wholesale price in Spain was EUR 283.19/MWh per megawatt-hour, which increased by 892.3% compared to last year. The increasing electricity prices have become a heavy burden on household and industrial users. On the other side, both the EU and the Spanish government proposed the policy goal of achieving affordable energy prices during the RE transition process [3,4,5]. Although Spain has reached its 2020 target to obtain 42% of its electricity from renewables [2,6], the current price situation shows that it has not achieved its affordable price policy target. Therefore, examining and detecting how to make Spain’s energy system provide a better RE transition with low energy prices is necessary. Some policy reforms might be essential. However, previous research has not engaged in answering these two questions systematically. Most of them focused on how the existing state-interventionist policies (high energy taxation, state subsidies, and state industrial access restrictions) conduct energy transition, not answering how the market process and entrepreneurship could bring more RE transition. As we discussed in the beginning, the ongoing high energy and electric prices show that current Spain’s energy policies have deficiencies, which should be improved differently.

In this regard, as previous interventionism-oriented energy policies have limited and probably reverse results, it is necessary to explore if any other alternative policy approaches could help the Spanish energy transition. We consider that a FME transition as an alternative policy approach could be a helping hand in answering the above two Spanish energy transition theses. It can challenge the traditional Spanish energy policy and make up for previous research gaps in not providing a free-market solution. Our earlier study on the EU’s free market energy transition supports this conception [7]. It found that reducing energy taxation, ceasing state subsidies, and eliminating state industrial access restrictions could offer more RE production and achieve relatively affordable energy and electricity prices. Therefore, we consider that it is necessary to apply its analytical framework for answering Spain’s energy transition thesis.

By applying the above FME approach, this paper synthesizes how state interventionist policies impede Spain’s energy transition. Our research aims to make up the above missing research gaps to provide a policy direction for the free-market energy transition. Our paper hypothesizes that high taxes, high government subsidies, and government industrial access restrictions breach private property rights, hindering Spain’s RE development. Our empirical results match the above assumptions. The above-mentioned three types of interventionist policies impede Spain’s energy transition goal of affordable prices, increasing the cost of the energy and power industries. The main findings are the following. (1) The current policy led to electricity prices remaining relatively high before and after the outbreak of the COVID-19 pandemic. Our calculations find that Spain’s household users’ electricity prices have an increasing tendency since 2009, while its industrial users’ prices started rising in 2019. (2) Hidden taxes have been a severe component of Spain’s electricity prices and are relatively high among the listed 28 IEA countries. Officially, Spain’s electricity tax component of its prices is relatively low among most of the IEA countries. The taxes are 5% for industrial users and 21% for houseful users [2]. However, our calculation shows that as of 2021, Spanish industrial and household electricity users must pay 54.4% and 48.5% taxation of the total electricity prices, respectively. (3) Even though the previous FIT-FIP systems as subsidy, taxation, and state industrial access institutions were abolished in 2012–2013, the Spanish government still conducts energy subsidies on electrification, coal–nuclear closure, and oil products [2]. The continuing interventionist policy impedes the energy and RE industries’ price coordination and market competition. (4) For state industrial access restrictions, 85% of energy production, 100% of the distribution network, and 90% of final sales are controlled by four giant firms (Endesa, Iberdrola, Naturgy, and EDP). Moreover, Spain fails the EU goal and Spanish *National Energy Poverty Strategy 2019–2024* to provide affordable energy prices as part of the green energy transition [3,4,5]. (5) Our data analysis shows that Spain is conducting energy transition positively regarding enhancing RE innovation and reducing greenhouse gas (GHG) emissions. However, as Spain’s household users’ electricity prices have been increasing since 2009 (while its industrial users’ prices started rising in 2019), the country has failed its policy target of providing affordable energy prices. In addition, we also use the ARIMA model to predict the possible trends of Spain’s electricity prices between 2023 and 2025. The modeling analysis shows that the prices for 2025 continue to be 4.6 times higher than in 2020. In other words, Spain will possibly fail the EU goal and its *National Energy Poverty Strategy 2019–2024* again to provide affordable energy prices as part of the green energy transition [3,4,5].

The study expects the following FME solutions for Spain’s energy transition to solve the above challenges: (1) continue to phrase out any interventionist general energy policy such as the FIT-FIP systems; (2) establish a market-based institution to hedge against the impact of market price fluctuations on the energy industry; (3) taxation, state subsidies, and state industrial access restrictions on energy and RE production should all be eliminated as much as possible; (4) enhance research on energy transition based on market forces; (5) conduct further free-market reform to create better energy and RE entrepreneurial innovation environment; and (6) adopt proper methods to face the energy crisis caused by the 2022 Russian invasion of Ukraine.

After all, we consider that this research’s results and FME approach policy are generalizable, at least inside the European institutions and other developed economies, as they already have the economic foundation and motivations for pursuing more environmental-friendly policy targets. In the first place, as we have mentioned above, our previous research on the European FME energy transition has studied the cases of Germany, Denmark, and the UK, showing that it is possible to apply the same framework to other European countries. Then, this paper extends the above scope into the case study of Spain. In the main texts of the paper, we will see that our hypotheses match with data analysis. In this regard, as free-market environmentalism is a new policy approach, European institutions and other developed economies may benefit positively from this policy direction, conducting energy transition with better quality and cheap prices.

The paper is structured as follows. Section 2 is the literature review. It aims to understand different energy policy approaches, reveals Spain’s current energy policies’ deficiencies, and discusses the potential FME energy policy for that country. In this section, we also provide a small debate on how to understand the merits and fallacies of a different policy approach based on free-market environmentalism. In this section, the related economic theories and their merits and deficiencies are also revealed and addressed. Section 3 is the empirical analysis. We compare the Spanish energy transition with Germany and the UK, showing whether Spain has been conducting energy transition successfully inside the EU. Since the Danish model is consistent with Germany, we use the German framework in our principal analysis. An ARIMA model is applied in Section 3 to clarify the economic performance of the Spanish electricity industry with a proposal for forecasting electricity prices. Section 4 is the research results. Section 5 proposes free-market environmental solutions and political reforms to facilitate Spain’s energy transition. Research limitations and possible future research directions are also presented in this section. Section 6 concludes.

## 2. Literature Review and Policy Debate: Why Spain Needs a Free-Market Energy Transition?

This section provides the literature review and policy debate. The literature review aims to understand different energy policy approaches, reveals Spain’s current energy policies’ deficiencies, and discusses the potential FME energy policy for that country, providing a foundation for the in-detailed analysis in the following few sections. In this section, the related economic theories are also revealed and addressed. Section 2.1 shows the essential theoretical tool that we use in this paper: the theory of free-market environmentalism. Section 2.2 is the introduction of Spain’s general energy transition. Section 2.3 to Section 2.5 separately different policy proposals. Specifically, we use free-market environmentalism as a benchmark to examine the literature thoroughly. Based on the above discussion, Section 2.6 overlooks different policy approaches for Spain’s energy policy, showing how to cover the current research gap.

### 2.1. The Theory of Free-Market Environmentalism

Any concrete public policy is ultimately based on assumptions that belong to a specific theory. This section introduces free-market environmentalism as our primary theoretical tool in this empirical study.

The theory of free-market environmentalism defines environmentalism as the science that studies human beings’ relations with each other and their environment [8]. Free-market environmentalism considers that the existing decentralized and spontaneous market process propelled by creative entrepreneurship coordinates better with and adjusts better for the rest of the species and elements of the natural environment than the centralized planned economy [8].

Free-market environmentalism considers that there are three fundamental problems with any centrally planned environmental policy. The three issues are also related to three economic theories. The first is the impossibility of economic calculation through centrally planned government policy. Proposed by Ludwig von Mises and the 1974 Nobel Prize in Economics winner Fredrich Hayek, the theory of the impossibility of centrally planned policy shows that human beings cannot act rationally when property rights are violated, as the necessary information and price signals are disturbed [9,10,11,12,13,14,15,16,17]. Identifying private property rights gives the property owner the incentive to protect the environment where he lives and sue anyone who violates his property’s environment. In contrast, the lack of private property generates the tragedy of the commons, and the environment is polluted without the incentive to protect it [9,10,11,12,13,14,15,16]. Therefore, even the most radical environmentalists cannot ensure that their centrally planned proposals would not cause even more environmental damage [8].

Secondly, nationalizing natural resources as public property prevents economic calculation and undermines entrepreneurship [8]. The theory of decision-making-featured entrepreneurship shows that as the market economy’s driving force [18,19], based on price signals, the entrepreneurs make better decisions and allocate resources more efficiently to protect the environment than the central planning of governments. However, it becomes impossible for enterprises to make economic calculations when natural resources are nationalized. Therefore, the related environmentally friendly products might not be produced due to the missing role of entrepreneurship.

Third, zero-sum games are created through public policies and legislative decisions, where the market might have better solved these problems. The public choice theory reveals that governmental orders substitute voluntary contracts and actions [8,20,21,22]. Voluntary negotiations might solve conflicts. However, as the 1974 Nobel Prize in Economics winner James Buchanan argues, the state legislature might cause the unexpected “one party wins, and the other loses” consequence [23]. In addition, incomprehensible legislation could cause the inefficiency of resource allocation through interventionism and regulation policies such as taxation, subsidies, and industry access restrictions. The result is that there is no way that the consumers and producers can internalize the costs and benefits of environmental protection-related production, and a zero-sum game is created by state legislation [24,25,26].

Previously, there was little theoretical and empirical literature on how property rights and the market promote RE’s development from free-market environmentalism [27,28]. Recently, based on free-market environmentalism, some literature has empirically shown how government intervention and regulation measures impede the RE market [29,30]. There are even some studies on Spain’s RE thesis [31,32]. Our previous research on the EU’s free-market energy transition in the case of Germany, Denmark, and the UK further concludes that state-interventionist policy as taxes, government subsidies, and industry access restrictions has impeded the energy transition in Germany, Denmark, and the UK. However, they are considered good examples of concluding free-market environmentalism in many policy approaches; although, they still have interventionalist policies impeding their RE transition [7].

In the literature on the history of European energy policy, both top-down and bottom-up approaches are two essential policy elements [33,34,35]. The former focuses on member states’ influence on designing and implementing policies at the EU level, and the latter relates to the European policies’ performance within the member states at a domestic level. Implementing a substantial public policy through a top-down or bottom-up initiative has many consequences at a decentralized level.

### 2.2. Introduction of Spain’s General Energy Transition

Spain’s overall energy strategy employs its “efficiency first” principle: (1) decoupling Spain’s economic growth depends on energy consumption and (2) reducing energy consumption [2,36]. In recent years, Spain has started achieving the above “efficiency first” policy; although, more actions are required to achieve the policy goals. Its energy intensity (the ratio of total consumption to gross domestic product) fell by 18% between 2008 and 2019 [2]. Its RE has expanded significantly in the last few decades. In terms of wind capacity in the first decade of the 21st century, Spain was the fourth country/region after the USA, Germany, and the Chinese Mainland [37].

Spain focuses on the following main energy policy targets: (1) massive development of RE, particularly solar and wind; (2) energy efficiency; (3) electrification; and (4) renewable hydrogen [2]. In addition, the Spanish government is concerned about the following economic effects that its energy–climate policies might bring about: (1) stimulate the economy; (2) create jobs; (3) modernize industry; (4) enhance competitiveness; (5) support vulnerable groups (i.e., promote employment opportunities in the energy transition, supported by a framework of vocational training, active labor policies, and support measures); (6) improve energy security; and (7) support research, development, and innovation [2].

Although the economic situation, such as employment rates in Spain, is relatively worse than in other West European countries [38], the Spanish state still prioritizes climate issues in its policy agenda. As a member of the EU, Spain is bound by the EU’s renewable and climate policies. In the middle run, Spain plans to achieve: (1) a 23% reduction in GHG emissions from 1990 levels; (2) a 42% share of renewables in energy end-use; (3) a 39.5% improvement in energy efficiency, and a 74% share of renewables in electricity generation before 2030 [2]. Spain plans to achieve 100% RE in electricity and 97% in the total energy mix in the long run in 2050 [2]. In recent years, the share of renewables (including non-renewable waste) has increased. The RE share in the national electricity mix grew from 24% in 2009 to 38% in 2019 [2]. Spain has achieved its 2020 target to source 42% of its electricity from renewables [2,6]. Likewise, the Spanish electricity energy market has also grown in recent years. According to the Spanish National Commission on Markets and Competition (CNMC), at the end of 2019, the number of consumers in Spanish retail electricity reached more than 29 million: 94% are domestic consumers (with a contracted power of less than 10 kW), and 6% correspond to more significant and industrial consumers [39]. Regarding consumption, 47% of the energy was consumed by industrial consumers, 28% by domestic consumers under 10 kW, and 25% by more significant domestic consumers [39].

However, electricity prices have been increasing in recent years [40], mainly due to the increasing energy and oil prices, the energy supply uncertainty during the post-pandemic, and the war in Ukraine [41,42]. The erupt energy demand and inflationary monetary policies are among the main factors of the increasing global energy prices [41,42]. In the case of Spain, *El País* shows that household electricity prices in October 2021 were 63% higher than a year earlier, according to Spanish National Statistics Institute (INE) [43]. On the side of industrial and agricultural uses, production costs have grown across all parameters for farming and breeding operations over 2020: electricity has gone up by 270%, tractor diesel by 73%, fertilizer by 48%, water by 33%, and seeds by 20% [43]. Industrial prices soar 31.9% in October 2021, the biggest rise in 45 years [1]. Data show that Spain’s price inflation in March 2022 fluctuates between 9% and 10%, up to 2.4 percentage points more than the previous month [44]. The increasing electricity prices have become a heavy burden on household and industrial users, making many (Truckers, farmers, auto and metal workers, hairdressers, and pensioners) on strikes or protests on the streets. It can be concluded that energy prices are strongly related to the development of Spain’s agricultural industry [45].

One of the main reasons why Spain’s electricity is relatively high among the EU countries is taxation on electricity. Although, as of 2019, Spain’s electricity tax component of its prices was relatively low among most of the IEA countries, which were 5% for industrial users and 21% for houseful users [2], the hidden tax has been a server component of why Spain’s electricity prices are relatively high among the listed 28 IEA countries. As of 2019, Spain has the eighth highest electricity prices for industrial uses (122.7 USD/MWh) and the fifth highest (287.7 USD/MWh) for household consumers. Due to the Spanish Official State Gazette’s Order IET/843/2012 [46] and Resolution of June 28 of 2012 [47], it is calculated that, as of 2015, although Spanish industrial users’ electricity tax component is only 3.87% of industrial users’ electricity prices, it does not include a 21% VAT, a 22.8% taxation for RE subsidies, a 4.79% taxation for the cost of transporting the electricity to the Balearic Islands and the Canary Islands, and an around 7.87% taxation component of the bill that is to pay the tariff deficit for closing nuclear power plants [48]. Therefore, data show that Spain’s industrial users had to pay altogether around 58% taxation of the whole electricity prices. In other words, only around 40% of the electricity prices that industrial consumers pay are the real cost of electricity.

High electricity prices have created social instability and harmed the Spanish economy. The RE’s regulation-interventionist policies become a considerable burden on the cost of agricultural production, so the related economic sectors might also be affected. For household electricity prices, the IEA indicates that Spain is also among the highest in IEA countries (fifth overall), at 287.7 USD/MWh, as data of 2019 show, with (officially admitted) taxes accounting for 21% [2]. The same pattern has been maintained in the last few years. The Spanish citizens must pay 35% “tolls and charges” (peajes y cargos in Spanish) plus 13.5% regular taxation and the 51.5% real energy cost [49]. Tolls refer to the cost of transport and distribution networks, and the charges refer to the premiums for renewables, extra-peninsular subsidies, and compensation for the tariff deficit [50]. In other words, Spanish citizens are required to pay 48.5% of total taxation for their energy consumption. As of March 2022, as other EU countries such as Denmark, Germany, and Portugal reduce their electricity prices, Spain still maintains its electricity price levels [51]. In this regard, compared to other EU countries, Spain keeps a high electricity taxation component. 

The above results show that Spain fails the EU goal and Spanish *National Energy Poverty Strategy 2019–2024* of providing affordable energy prices as a part of the green energy transition [3,4,5]. It also failed Spain’s goal of making its electricity supply at the “lowest possible” cost based on its electricity privatization process since 1997 due to Law 54/1997 [5,52]. Moreover, the coal phase-out is well on track. Coal only provides around 5% of electricity generation in 2019 and even less in 2020 [2].

Another controversy in Spain’s energy issue is the reduction in nuclear power. Nuclear power, as an essential source of low-carbon generation due to IEA and International Atomic Energy Agency (IAEA) [53,54], accounted for 22% of Spain’s power generation in 2019 [2], almost one-fourth of the country’s power generation. As a method to reduce greenhouse emissions (GHS) and achieve climate protection, nuclear power has been gradually phased out in many developed countries after the 2011 Fukushima nuclear disaster, including Spain [2]. However, the Spanish state decided to shut it down in 2027, and four of Spain’s seven nuclear reactors are scheduled to close by the end of 2030 (they represent around 4 gigawatts of capacity) [2]. It is argued that reducing nuclear dependence is one of the causes of increasing electricity prices [55]. The IEA is concerned that the rapid closure of coal and nuclear facilities over the coming decade might increase Spain’s demand for natural gas, especially if new renewables capacity cannot be built as quickly as planned [2]. From the FME approach, this closure is through government legislation and central planning. Entrepreneurship, as the driving force of the market economy, might also be disincentivized, which is not favorable for Spain’s long-run energy transition. One example is that Spain is delaying interconnecting its RE electricity projects with France due to the two states’ discoordination. The IEA even warns that it may cause Spain to fall short of its EU interconnectivity targets of 10% by 2020 and its 15% target by 2030 [2]. Positively, the EU has also listed natural gas and nuclear power as clean energy since February 2022 [56]. They could be alternative energy options for Spain instead of more state-interventionist policies.

Although Spain is considered one of the EU countries achieving more RE, it still must improve and reduce its reliance on fossil fuels, meeting its targets for renewables penetration and decarbonization. A previous study discovered that “only 18% of Spanish households consume electricity and natural gas, which are only available in major cities in Spain due to the limited development of pipeline distribution” [57]. In addition, fuel use in Spanish cities and towns is uneven, as “households living in towns with fewer than 10,000 inhabitants show a share of fuel over total expenditure 34.6% greater than households living in cities over 500,000 inhabitants”. In contrast, “the expenditure share on public transport is 61% lower” [57]. Moreover, the data of IEA indicate that Spain has a huge demand for, and supply of, fossil fuel energy. As of 2019, most of Spain’s energy supply and demand were met with fossil fuels, which accounted for 72% of total energy supply and 68% of total final consumption in 2019 [2]. However, as two-thirds of Spain’s total energy supply was produced domestically in 2019 [2], Spain heavily depends on importing foreign countries’ fossil fuels. The above data show that Spain still has a long way to go to handle its fuel energy issue. The IEA is concerned that Spain’s total energy mix is still heavily dominated by fossil fuels in the electricity sector, especially the transport, industry, and buildings sectors [2,36].

The IEA suggests that Spain consider changes to its taxation system (notably incorporating the cost of carbon into end-use prices), reducing barriers to increased uptake of clean electricity in more end uses [2]. Previous studies based on free-market environmentalism also suggest that reducing and eliminating industrial access restrictions promote more uses of RE [7]. These studies pointed out that interventionist policy distorts the energy transition process, as entrepreneurship and its innovation might be disincentivized. They are also concerned that the deregulation approach might help reduce electricity prices while the production of renewable is increasing. This conclusion also concurs with the IEA’s further RE policy suggestions on eliminating tax barriers for RE [58,59,60].

### 2.3. Literature Review on Spain’s Energy Policy: Pro-Interventionism

There are many other studies on Spanish energy and its price-related issues. However, their conclusions are verified and contradicted in the same survey. Abdelradi and Serra discovered an empirical correlation between the prices of Spanish sunflower oil and the biodiesel industry’s production [45]. Mendiluce et al. correctly pointed out that countries such as Germany have been doing better in RE production and innovation [36]. However, they did not mention an essential question about the success of Germany’s energy transition: whether it is through state intervention or a market-oriented approach? Their research proposed a contradicted view. On the one hand, the state should remove “subsidies to inefficient industries”. On the other hand, it proposed state-interventionist policies using subsidies and price signals to incentivize energy-efficient investments, fuel taxation, and traffic regulations [36]. The essentials of private property rights and entrepreneurship are neglected in their energy studies. Despite the research deficiency, we believe that their proposal to develop workforce capacity for energy saving in buildings and to educate society in an energy efficiency culture is positive. It could also be executed based on FME principles.

Labandeira et al. analyzed the economic, environmental, and distribution effects of green taxes in Spain, proposing a further green tax to reduce CO_2_ emission [57]. Montoya et al. proposed more regulation policies to incentivize the Spanish energy industry [61]. They argue that the change in market activities might cause an increase in costs in the short run. However, if other conditions remain the same, the market will provide more options for energy consumers in the future. Therefore, the increased supply will reduce costs in the middle or long run. They treat the market as a given framework to maximize resources, failing to understand that the market is a dynamic process. In short, the above empirical study does not count and discuss the potential and unseen entrepreneurial innovation impeded by taxation, showing that this paper has massive epistemological and methodological deficiencies. The impossibility of a centrally planned economy, the principles of private property rights, and entrepreneurship as the driving force of the market economy are all overlooked. On the contrary, Montoya et al. recognized that state regulation causes difficulties in the wind energy industry, as “Spanish wind energy is subject to environmental legislation that limits it by forbidding installation in national and natural parks” [61].

Burke and Stephens proposed the thesis of energy democracy globally, proposing the importance of local and small enterprises’ participation during the energy transition process [62]. They also opposed the centralization of energy policy decision making and significant energy cooperation with oligarchical market shares. However, they objected to privatization and capitalization, disregarding the functions of the market, private property rights, entrepreneurship, and price coordination.

### 2.4. Literature Review on Spain’s Energy Policy: Pro-Market

Contrary to the above interventionist view, scholars from management science have a different viewpoint. Heras-Saizarbitoria et al. affirmed the positive RE results of a decentralized energy industry [63]. They criticized the Spanish states for only authorizing energy business management into the hands of five-big energy companies (Iberdrola, Endesa, Naturgy, Fenosa, and Repsol), creating a state-supported industrial oligarchy. They also stressed that the appearance of the five-big RE cooperatives in Spain might have caused citizens’ dissatisfaction with energy. Moreover, they pointed out that small energy enterprises with better RE innovation are squeezed out of the Spanish energy market due to the Spanish state industrial access restrictions. Their approaches concur with the FME approach, as they stressed the relationship between the state industrial access restrictions and the poor RE market competition. More importantly, the studies from management science have rectified the literatures’ deficiencies that supported state intervenient RE policies, overlooking the real world of RE’s entrepreneurial organizations. However, although the management scholars’ studies have the above merits, they do not systematically analyze the other interventionist policies such as taxation and state subsidies, making further studies imperative.

Lesser and Xu recognized that market prices could enhance energy efficiency and reduce consumer costs. However, they still supported regulated feed-in tariffs (FIT) prices [64]. Langniß et al. introduced three pro-market models that might be constrictive for energy transition [65]. Among them is the Optional Bonus Model (the model has been renewed annually or monthly through energy suppliers’ preferred remuneration with a minimum price floor) that Spain adopted between 2005 and 2006. However, the authors also pointed out that this fixed price does not benefit the Spanish energy consumer as they must pay higher costs. Therefore, although the above scholars tried to design a more pro-market FIT system that could enhance the energy industry’s competitiveness and reduce energy consumers’ costs, they all admitted that these plans have shortcomings and could not entirely reflect the dynamic energy market, making planning impossible. Ciarreta et al. pointed out that the reform in 2013 and 2014 abolished FIT and feed-in premiums (FIP) systems while maintaining state price subsidies for specific energy production [5].

### 2.5. Literature Review on the Proposals of Spain’s Free-Market Energy Transition

Recently, free-market environmentalism-based studies were applied to analyze the Spanish energy transition and its state-interventionist components. One of our previous studies pointed out that, on the one hand, the Spanish government regulation and premiums have raised legal barriers to entry, strengthening market concentration in four giant firms [32]. On the other hand, the close dependence of renewables on government aid has led to rent-seeking behavior, which consumers must finance with higher electricity bills [32]. Peña-Ramos et al. also showed the recent failure of the Spanish government’s effort to trade and buy wind energy from the Euro-Mediterranean countries such as Morocco, France, and Portugal [31]. It was pointed out that the states centrally planned coordination was too complicated and made the trading deal impossible. On another side, no previous literature has thoroughly studied the Spanish energy system from an FME perspective.

In contrast, international market-based RE energy trading might be a solution to help Spain attain more wind energy. Moreover, having the same conclusion as IEA [2], they consider that embracing natural gas is a medium-term method to achieve a 100% RE in the future. They also pointed out that other conventional backup sources such as coal or nuclear might also be used to keep electricity prices low. From a FME perspective, the market solution could solve the discoordination that central state planning caused. While during the transition period, the formulation of energy policies should respect the actual price affordability of consumers and enterprises (which is also one of the EU and Spain’s energy transition goals, as we showed above [3,4,5]) instead of forcing consumers and enterprises to use RE products for the energy transition. The government’s forcible intervention in the market may lead to artificially high prices. Still, the costs could also be passed on to consumers and enterprises, affecting economic development and people’s living standards. This is contrary to Spain and the EU’s policy of affordable energy prices [3,4,5].

### 2.6. An Overlook of Different Policy Approach: How to Cover the Research Gap?

Table 1 below lists the main points and deficiencies of various policy proposals on Spain’s energy industry. The Table helps us overlook different policy directions and lets us perceive what the FME approach can do to improve Spain’s energy policy. As these policy points have been addressed in the above sections, in this section, we emphasize how to cover the current research gap.

For the pro-interventionist policy proposals, they supported subsidies, taxations, and government planning on Spain’s energy industry [45,57,61,62]. At the same time, the roles of private property rights, market price coordination, and entrepreneurship in Spanish RE development have been overlooked. These studies did not cover the adverse effects of these policies on Spanish RE production. Hence, the FME approach should analyze these side effects of the above interventionist policy proposals.

The pro-market policy proposals [63,64,65] admitted that the functions of the above market institutions have been positive for the Spanish RE industry. However, they did study the negative consequences of taxation and state subsidies on Spain’s energy transition, having a reserved view on a pure market pricing mechanism. Therefore, it is indispensable for the FME approach to discuss this reserved opinion.

Previously, there was little theoretical and empirical literature on how property rights and the market promote RE’s development from free-market environmentalism [27,28]. As most countries have adopted interventionist energy policies, this policy direction should be applied more in practice. Secondly, there is a lack of communication between FME proposals and policymakers. The influence of the FME approach is limited to very small academic and policy-making cycles. In this regard, it is necessary to make the FME proposal visible in the policy-decision process.

Moreover, no previous literature has thoroughly and systematically studied the Spanish energy system from an FME perspective. Previous FME studies on the Spanish energy system should have connected more with the pro-market policy proposals supported by authorities such as the IEA and the IAEA. In this regard, the FME policy for Spain needs to study more policy issues in detail to cover more research gaps. Topics such as human capital use in RE transition [66], RE firms’ trading behaviors [67], water sustainability [68], hydrological cycle [69] (considering that Spain has dry weather due to the Mediterranean climate), market-based energy trading and pricing [70] should be considered by the FME studies.

**Table 1:** Main points and deficiencies of different policy approach to Spain’s energy industry.

	Main Points	Deficiencies and Problems
**Free-Market** **Environmentalism**	(1) The theory of the impossibility of a centrally planned economy shows that identifying private property rights gives the property owner the incentive to protect the environment [9,10,11,12,13,14,15,16]. (2) The decision-making theory featuring entrepreneurship shows that nationalizing natural resources as public property prevents economic calculation and undermines entrepreneurship [8,18,19]. (3) The public choice theory reveals that zero-sum games are created through public policies and legislative decisions, where the market might have solved these problems better [23].	(1) There was little theoretical and empirical literature on how property rights and the market promote RE’s development from free-market environmentalism [27,28]. (2) As most countries adopt interventionist energy policies, this policy direction should be applied more in practice. (3) There is a lack of communication between FME proposals and policymakers. The influence of the FME approach is limited to very small academic and policy-making cycles.
**Pro-Interventionism**	(1) Abdelradi and Serra discovered a correlation between the prices of Spanish sunflower oil and the biodiesel industry’s production [45]. They support state subsidies on certain energy investments. (2) Labandeira et al. analyzed the economic, environmental, and distribution effects of green tax in Spain, proposing a more green tax to reduce CO_2_ emissions [57]. Montoya et al. proposed more regulation policies to incentivize the Spanish energy industry [61]. (3) Burke and Stephens proposed the thesis of energy democracy globally and supported small energy enterprises’ participation [62]. They also opposed the energy market’s privatization and capitalization and energy policy’s centralization of decision making and oligarchical market shares.	(1) Abdelradi and Serra proposed a contradicted view. They supported the removal of certain energy subsidies while supporting others. The essentials of private property rights and entrepreneurship in industrial development are neglected. (2) Labandeira et al. and Montoya et al. treated the market as a given framework to maximize resources, failing to understand that the market is a dynamic process. They do not count and discuss the potential and unseen RE industry’s entrepreneurial innovation impeded by taxation. (3) Burke and Stephens’ objection to privatization and capitalization of the energy market disregarded the functions of the market, private property rights, entrepreneurship, and price coordination.
**Pro-Market**	(1) Heras-Saizarbitoria et al. showed the positive RE results of a decentralized energy industry with small energy firms’ innovation, criticizing the Spanish state’s oligarchical energy market [63]. (2) Lesser and Xu recognized that market prices could enhance energy efficiency and reduce consumer costs while supporting specific fixed prices [64]. (3) Langniß et al. introduced three pro-market models that might be constrictive for energy transition [65].	(1) Heras-Saizarbitoria et al. did not systematically analyze the other interventionist policies, such as taxation and state subsidies, making further studies imperative. (2) Lesser and Xu still supported regulated FIT prices while considering that market price signals could work more efficiently. (3) Langniß et al. admitted their fixed price plans might not benefit the Spanish energy consumers as they must pay higher costs.
**Spain’s Free-Market Energy Transition**	(1) Espinosa et al. found that the Spanish government regulation and premiums have raised legal barriers to entry, strengthening market concentration in four giant firms [32]. (2) Peña-Ramos et al. showed the recent failure of the Spanish government’s effort to trade and buy wind energy from the Euro-Mediterranean countries [31]. (3) IEA considers that embracing natural gas is a medium-term method to achieve a 100% RE in the future [2]. The EU has also listed natural gas and nuclear power as clean energy since February 2022 [56]. Nuclear power is an essential source of low-carbon generation due to IEA and IAEA [53,54].	(1) No previous literature has thoroughly and systematically studied the Spanish energy system from an FME perspective. (2) Previous FME studies on the Spanish energy system should connect more with the pro-market policy proposals supported by authorities such as the IEA and the IAEA. (3) FME policy for Spain needs to study more policy issues in detail to cover more research gaps. Topics such as human capital use in RE transition [66], RE firms’ trading behaviors [67], water sustainability [68], hydrological cycle [69] (considering that Spain has dry weather due to the Mediterranean climate), market-based energy trading and pricing [70] should be considered by the FME studies.

**Figure 1:**
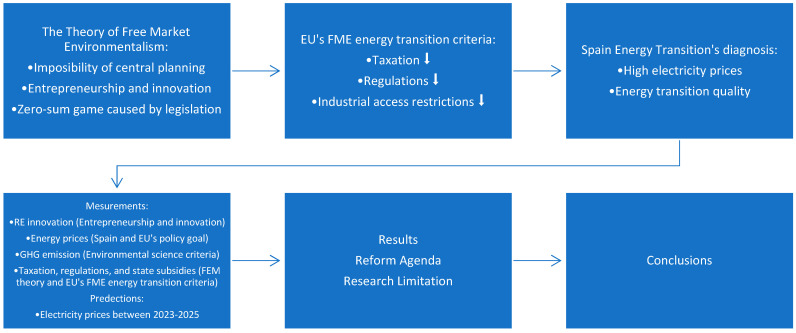
Research framework.

Our research intends to compensate for this gap by synthetically analyzing the Spanish electricity price thesis and its energy policies on taxation, state subsidies, and industrial access regulations. Figure 1 below shows the research framework of this paper. Based on the three theoretical pillows of the FME theory [8] that we have discussed above, our previous research [7] provided three policy criteria for EU’s FME energy transition with affordable emery prices: reducing the taxation, regulations, and industrial access restrictions on the energy industry. This policy criterion provides us propositions to diagnose the Spanish transition thesis: how to solve its high and increasing electricity prices problem with a better RE transition.

Therefore, four measurements are provided to measure Spain’s energy transition in the same framework as our previous research [7] on the EU’s FME energy transition. (1) The first is the impact on RE innovation, measured through the increases in the share of RE in total gross available energy, the rise in the share of RE in total gross electricity production, and the number of jobs in RE. It applies the FME approach’s entrepreneurship theory, which argues that entrepreneurship is the driving force of the market economy and energy innovation [18,19]. (2) The second is the impact on energy prices. It is measured through the evolution of the electricity prices for household and non-household (industrial) consumers. This measurement employs Spain and the EU’s policy of providing affordable energy prices [3,4,5]. (3) The third is the impact on GHG emissions, measured as the evolution of the number of emissions over time. This measurement applies the criteria of environmental science [71]. (4) The impact of taxation, state subsidies, and state industry access restrictions on the development of RE and electricity markets and their prices. This category is measured through the FEM theory [8] and EU’s FME energy transition criteria [7]. Moreover, an ARIMA econometric model is provided to predict Spain’s electricity prices between 2023 and 2025. We start the data analysis in the next section based on the above measurements.

## 3. Data Analysis and Method

This section deals with the data analysis of Spain’s energy transition. Section 3.1 provides a statistical analysis of whether Spain’s energy transition is successful. Section 3.2 uses the Box–Jenkins methodology, or ARIMA econometric model, to predict electricity prices in Spain under current circumstances. We also compare the Spanish energy transition with Germany and the UK, showing whether Spain has been conducting energy transition successfully inside the EU.

### 3.1. Measured Statistic Parameters: A Comparison with Germany and the United Kingdom’s RE Transition

The parameters on which we evaluate the RE transition continue the framework we used in our previous study of the RE transition in Denmark, Germany, and the UK [7]. The parameters are the following. (1) The impact on RE innovation, measured through the increases in the share of RE in total gross available energy, the rise in the share of RE in total gross electricity production, and the number of jobs in RE. (2) The impact on energy prices, measured through the evolution of the electricity prices for household and non-household (industrial) consumers. (3) The impact on GHG emissions, measured as the evolution of the number of emissions over time. (4) The impact of taxation, state subsidies, and state industry access restrictions on the development of RE and electricity markets along with their prices. These four degrees reflect different aspects of any RE transition from the current European environmental policy perspective mentioned above: lower prices, more RE, and less GHG emissions.

**Figure 2:**
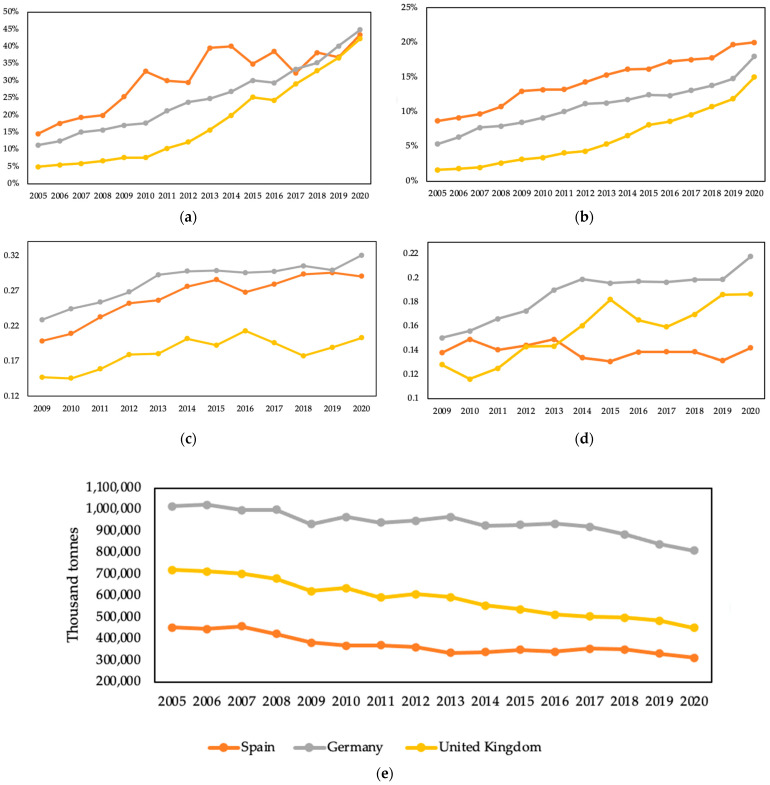
Evolution of variables related to the RE transition in Spain from 2005 to 2020, compared with Germany and the UK (source: adapted from [7] and own calculations based on Eurostat data [72]): (**a**) Share of RE in gross electricity production. (**b**) Share of RE in gross available energy. (**c**) Price of electricity for household (all taxes and levies included in constant prices of 2015 (EUR per kWh). (**d**) Price of electricity for the non-household consumer (all taxes and levies included in constant prices of 2015 (EUR per kWh). (**e**) GHG emissions, 1990–2018.

Figure 2 comprises five graphs showing the evolution of the parameters we propose for analyzing the RE transition in Spain from 2005 to 2020 (the post-pandemic era). These variables include the share of RE in gross electricity production, the percentage of RE in gross available energy, electricity prices for households and non-household consumers (all taxes and levies included), and GHG emissions. Based on our previous study on the EU’s free-market energy transition [7], we also compare the data with Germany and the UK in the figure to show the trends of Spain’s energy transition. As Demark has similar local initiatives and high electricity taxation patterns [7], we only choose Germany as the sampled case in this paper to simplify the comparison.

The recent rapid increase in energy and electricity prices during the post-pandemic era is analyzed separately as the aforementioned analytical conditions have changed a lot since the COVID-19 outbreak. Although our previous study [7] involves the energy transition of Germany and the UK since 1990, due to data accessibility, we only choose the period since 2005 for the study of Spain. The electricity prices have been selected from 2009 to 2020 are shown in Figure 2.

At first glance from Figure 2, it seems that in 15 years, from 2005 to 2020, Spain has positive features in their respective RE transitions. This tendency is especially evident in the increasing trends in the share of RE in gross electricity production (Figure 2a), gross available energy (Figure 2b), and the decreasing trend in GHG emissions since 2005 (Figure 2e). However, as the supply of RE increased, the price of electricity (Figure 2c,d) did not show a corresponding downward trend. In Figure 2c, Spain’s household electricity prices are increasing like Germany’s. Although in Figure 2d, its non-household consumer prices have remained at a relatively low level from 2009 to 2020, even the electricity prices for the non-household consumers dropped from 2010 (0.1493 EUR per kWh) to 2015 (0.131 EUR per kWh). However, the prices started to have a slight upward trend in 2015. As we have mentioned in Section 2.2, due to the pandemic outbreak, energy shortage factors, and general price inflation trend factors, along with the others, Spain does not fulfill the EU and its own goal of having an affordable energy price. This result may alter the traditional view of Spain’s energy transitions that it is doing well on RE transition [2]; although, Figure 2a–c shows a positive result. Therefore, conducting an in-depth analysis of Spain’s RE markets, electricity prices, and relationships with free-market environmentalism is necessary.

Table 2 comprises our analysis of Spain’s RE transition from the perspective of free-market environmentalism and the indicators we have selected to measure Spain’s transition’s progress. Table 2 contains an overview of Spain’s energy system, the key policies implemented by the country, and the taxes and subsidies introduced between 1990 and 2018. There is also a quantitative part with data corresponding to the selected variables. Most of the figures are our calculations based on Eurostat, the European Environment Agency, and EurObserv’ER. The statistics allow us to measure Spain’s progress in the RE transition and the impact of its policies and institutional frameworks. Table 2 shows that Germany has provided spaces for the local community and firm-level initiatives to conduct energy transition compared to Spain. At the same time, the UK has the experience of low electricity taxation of 5% with the lowest household electricity prices compared with the other two sampled countries. This has reduced energy costs and has enhanced a better and more innovative RE transition.

We focus on innovation, GHG emissions, and electricity prices because they reflect three aspects usually addressed in energy policy literature, as we have mentioned above. Additionally, they are priorities for the current European environmental policy. First, the impact on innovation is measured by increasing the share of RE in total gross available energy, the increase in the percentage of RE in total gross electricity production, and the number of jobs in RE in 2020. Second, the increases in the two shares are calculated as the rate of change in RE shares in their respective totals between 2005 and 2020.

For their part, jobs are expressed in absolute terms, and we have calculated the employment/total population ratio to compare the figures between the countries. The GHG emissions impact is calculated as the rate change between 2005 and 2020. The impact on electricity prices is also measured as the change in electricity prices for household and non-household consumers between 2009 and 2020. Finally, we provide nominal prices to compare data in real terms without price inflation.

Looking first at Spain’s policies and institutional design outcomes, we can make the following comments. First, regarding the impact on innovation, Spain has considerably increased the share of RE in gross available energy and gross electricity production. The former increased by 11.6% and the latter by 29% since 2005 [59]. Second, the impact on innovation has also resulted in Spain’s job creation, with more than 950,809 jobs created by activities related to renewable energies as of 2020 [59]. These jobs created represent a rate of 0.002008204 over the total population. Although Spain has suffered from the outbreak of the COVID-19 pandemic, these data are higher than Germany and the UK in job creation associated with RE. Third, Spain has reduced its GHG emissions by 30.7% from 2005 to 2020. Thus, it evidences the substantial efforts made to reduce GHG emissions. However, despite a positive result, Spain is still far from meeting the target set in the Climate Action Plan 2050 of a 40% cut in GHG emissions by 2020, taking 1990 as the base year [59].

Spain’s electricity prices have experienced an increase from 2009 to 2020 both for household and non-household consumers: 2.9% (for households) and 46.6% (for non-household) [59]. Hence, Spain’s non-household consumer prices have remained relatively low from 2009 to 2020; even the electricity prices for the non-household consumers have dropped from 2010 (0.1493 EUR per kWh) to 2015 (0.131 EUR per kWh). However, the prices started to have a slight upward trend in 2015. As mentioned, Spain does not fulfill the EU’s goal of affordable energy prices due to the pandemic outbreak, energy shortage, a general price inflation trend, and the war in Ukraine. This result can alter the traditional view of Spain’s energy transitions that it is doing positively on RE transition.

**Table 2:** Spain’s energy industry systems and policies (compared with Germany and the UK), 2005–2020.

	Spain	Germany	The UK
Energy system	A mixed decision-making system. The central government plays a more decision-making role in energy policy than the autonomous communities. A top-down state planning and state funding institution are missing the functions of bottom-up entrepreneurial initiatives.	A decentralized system with considerable government and civil society involvement in the energy transition. A combination of top-down and bottom-up actions.	Hypothetically the most market-oriented policymaking lacks bottom-up initiatives. Instead, the top-down processes play an essential role through market-oriented policies—more government intervention over time.
Essential energy policies	State research institutions have conducted main energy R&D policy and guidance since 2007 [4,73]. Support enhances economic development and job creation through RE innovation [73]. There is a strong focus on green hydrogen, energy storage, and RE innovation in mobility and industry [4]. The FIT-FIP systems (1997–2012).The liberalization of the electricity market (1998).	Support for wind energy through FIT and tax breaks. Solar PV supports through investment subsidies, low-interest loans, and FIT [57]. FIT system (1992–present). The liberalization of the electricity market (1998).	A national program to support R&D started in 1975 [22]. Liberalization of the electricity market (1989). Tendering system NFFO (1990–2002). RO system, green certificates (2002–present). FIT system (2010–present).
Taxation, state industry access restrictions, and state subsidies	A high electricity tax is more than 50% (see Section 4.2). State subsidy-taxation (FIT) and price control (FIP) [74] till 2012–2013. High hidden taxation on electricity. Subsidies for electrical products, oil, and nuclear closure in the current institutions, while coal subsidies ceased in 2018 [2]. Decades of strong state access restrictions in energy and RE industries [32].	A high electricity tax is more than 60% [7]. At different rates, energy tax on oil products, natural gas, and coal and coke products. Several tax concessions (e.g., heating fuels, electricity in manufacturing industries, and agriculture). Biofuels are subsidized through the EU biofuels targets. Carbon tax for emissions in non-ETS sectors. Surcharge for consumer electricity bills to pay for renewables subsidies. The high share of costs onto households [58].	Low electricity tax as 5% [7]. Levy control framework (LFC) for low-carbon electricity costs levied on consumers’ bills (it covers electricity only). In 2017, the Control for Low Carbon Levies replaced the LFC. Climate Change Levy (2001) levied energy supply to business and public sector consumers [60].
Impact on innovation			
Share of renewables in gross electricity production	14.6% (2005) 43.46% (2020) Δ% = 29%	11.32% (2005) 44.9% (2020) Δ% = 34%	4.99% (2005) 42.3% (2020) Δ% = 37%
Share of renewables in gross available energy	8.5% (2005) 20% (2020) Δ = 11.6%	6.7% (2005) 18% (2020) Δ = 11.3%	1.3 (2005) 15% (2020) Δ = 13.7%
Jobs created (as of 2020)	950,809	121,700	120,400
Total jobs created/pop (as of 2020)	0.002008204	0.001452546	0.001773561
Impact on GHG emissions (2005–2020)	−30.7%	−20.3%	−37.2%
Impact on electricity prices			
Non-household consumer prices (2009–2020)	2.9%	44.9%	45.6%
Household consumer prices	46.6%	40.2%	50.3%

Source: Own elaboration based on IEA, Eurostat, European Environment Agency (EEA).

### 3.2. Model Analysis of Spain’s Electricity Prices

After analyzing the effects of government interventionism on energy costs and prices in Spain compared to its history and Germany and the UK, this section uses an econometric model to forecast the electricity price to 2025. The use of models to predict observations ordered in time is well-known as a time series. The central assumption of time series forecasting is that the past conditions that determine a variable or set of variables will remain unchanged in the future. Gujarati et al. [75] point out five approaches to forecasting based on time series: (i) exponential smoothing methods, (ii) single-equation regression models, (iii) simultaneous-equation regression models, (iv) autoregressive integrated moving average (ARIMA) models, and (v) vector autoregressive (VAR) models.

We use the ARIMA methodology, which comes from the following elements [76]:

- An autoregressive process (AR) is a random process for general cases of variability over time.
- Integrated (I): a time series is integrated of order 1, that is I (1), if its first differences are stationary. Similarly, if a time series is I (2), its second difference is stationary. Therefore, if a time series must be differentiated d times to make it static and then apply a weapon model (p, q), the series is said to be an ARIMA (p, d, q), that is, an integrated moving average autoregressive time series. Where p is the autoregressive terms, q is the moving average terms, and d is the number of times the time series must be differenced to make it stationary.
- Moving Average (MA): Calculated to smooth the volatility of a data series by averaging subsets of the entire series.

ARIMA emphasizes the analysis of probabilistic or stochastic properties of time series. Unlike other regression models, in which Yt is predicted by the k regressors X1, X2, X3, … Xk, in Box–Jenkins models, Yt can be explained by past or lagged data and by the coefficients or stochastic error terms. Most time series are stationary because the mean and variance are constant, and the covariance is time-invariant. Most economic series are not stationary, so it is necessary to differentiate them. The advantages of the ARIMA model are the following:

- Straightforward methodology, where mathematical and statistical processes are included.
- The empirical application that these models have for sample situations.
- These models have been demonstrated to bring efficiency in predicting the series in the short term.

**Figure 3:**
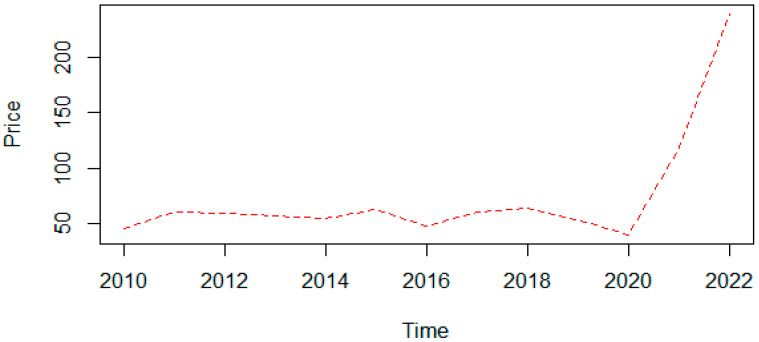
The monthly price of electricity in Spain (in euros per megawatt-hour). Source: Own elaboration from Statista and Rstudio [77].

**Figure 4:**
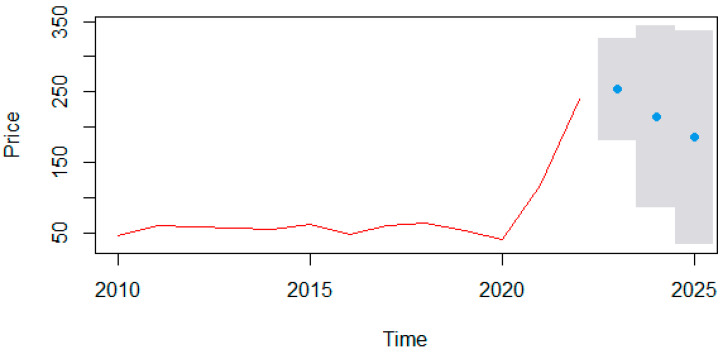
Forecast from ARIMA (1.0.1) between 2023 and 2025 (in euros per megawatt-hour). Source: Own elaboration from Statista and Rstudio [77].

We use the statistical software R Studio to build the ARIMA model in three main steps. First, we operate the “ts” command to construct a time series on the final average price of electricity in Spain from 2010 to 2022 (in euros per megawatt-hour). The source is the Statista database, the statistics portal for European economic data [77]. Figure 3 shows that the price of electricity in Spain multiplied by six from EUR 45 in 2010 to EUR 270 in 2022. Moreover, it indicates a standard deviation of 8 between 2010 and 2020 and 120 between 2020 and 2022. Second, we use the “auto.arima” command to conduct automatic tests until we find the best model according to our data. R Studio proposes the ARIMA (1,0,1) model with a non-zero mean that has a lag in the autoregressive process. It does not require differentiation to become stationary, and requires a moving average (Table 3). Finally, we use the forecast command to forecast the electricity price in euros per megawatt-hour for 2023, 2024, and 2025 in Spain with 95 percent confidence. Figure 4 shows that the electricity price in 2023 will be EUR 255 within EUR 182 and 325. Then, the electricity price in 2024 will be EUR 215 within EUR 85 and 350, and the electricity price in 2025 will be EUR 186 within EUR 34 and 340.

The result of the ARIMA model supports our hypothesis that high taxes, high government subsidies, and government industrial access restrictions breach private property rights, contributing to the rise in electricity costs and prices in Spain. Other things being equal, maintaining the current government intervention in the industry implies an electricity price increase. The uncertainty about the new policies of the Spanish government and the international context in the performance of the renewable energy industry denotes a wide range of possibilities between 2023 and 2025. The upward tendency of the time series seems to soften, but the prices for 2025 continue to be 4.6 times higher than 2020. In other words, our modeling results match our statistic results in Section 3.2, showing that Spain will fail the EU goal and Spanish National Energy Poverty Strategy 2019–2024 to provide affordable energy prices as part of the green energy transition.

The advantage of an ARIMA model is that it does not require other data series referring to the same period and saves the identification and specification of the model by applying traditional econometrics. It should be added that the increase in electricity market interventions imposed in the last decade by the Spanish government introduced volatility to price signals because artificial costs created did not obey any market variable, and that would be impossible to calculate and predict with any model. This uncertainty adds greater complexity to any forecast study on electricity prices. This section showed that Spain is doing positive work in RE innovation and job creation and has significantly reduced its GHG emissions in recent years. However, it has an increasing trend in electricity prices. Our model analysis also suggested that Spain may soon have a growing electricity price. Why is Spain’s energy and electricity expensive and has an ascending trend? The following section shows how state-interventionist policies distort Spain’s energy transition and electricity prices.

## 4. Results: A State Interventionist RE Institutions with Mixed Decision-Making Features

This section analyzes Spain’s state-interventionist policies’ consequences on the electricity price. Section 4.1 and Section 4.2 show Spain’s energy policies are conducted between state intervention energy policies with a mixed decision-making feature. From Section 4.3 to Section 4.4, we analyze Spain’s general energy policies, taxations, state subsidies, and industrial access restrictions, which are vital factors related to Spain’s energy prices due to our studies.

### 4.1. Top-Down Initiatives and a Mixed Energy Transition Result

The above empirical results show that Spain’s electricity prices have increased. However, due to the increasing energy and oil prices and the uncertainty of energy supply during the post-pandemic era [41,42], the erupting energy demand and inflationary monetary policies are among the main factors causing the increasing global energy prices [41,42]. Household electricity prices in October 2021 were 63% higher than a year earlier, according to Spanish National Statistics Institute (INE) [43]. On the side of industrial and agricultural uses, production costs have grown across all parameters for farming and breeding operations over 2020: electricity has gone up by 270%, tractor diesel by 73%, fertilizer by 48%, water by 33%, and seeds by 20% [43]. Industrial prices soared 31.9% in October 2021, the most significant rise in 45 years [1]. These increasing electricity prices have become a heavy burden on household and industrial users, making many (truckers, farmers, auto and metal workers, hairdressers, and pensioners) attend street strikes or protests [43]. Therefore, if the RE’s regulation-interventionist policies become a considerable burden on the cost of agricultural production, the related economic sectors might also be affected. High electricity prices have created social instability and harmed the Spanish economy.

For household electricity prices, the IEA indicates that Spain is also among the highest in IEA countries (fifth overall), at 287.7 USD/MWh, as data of 2019 show, with officially admitted taxes accounting for 21% [2]. The same pattern has been maintained in the last few years. Spanish citizens should pay 48.5% of total taxation for their energy consumption. Considering these results, we can affirm that Spain is making the RE transition well; although, it needs to increase the rate to reduce GHG emissions. The country also has a problem with rising electricity prices, especially for non-household users. Spain is not fulfilling one of the objectives of any energy transition, namely, to accomplish it at an affordable cost [3].

Spain’s energy system mixes the central government’s planning and the decision making of its 17 autonomous communities. The former institution plays a more decision-making role in energy policy, responsible for general taxation (such as VAT and different forms of national energy tax), energy subsidies, energy industrial access restrictions, and other state-interventionist policies [2,57,61]. Therefore, as shown above, Spain has top-down state planning in energy policymaking. On the contrary, the latter autonomous institutions have limited roles, responsible for authorizing certain power plants and energy networks [2]. However, autonomous communities have minor decision-making roles since the central government has the power to decide the general taxation level and other policies that may influence the development of the energy industry in each autonomous community.

Spain started its electricity market liberalization in 1998 under Premier Jose Maria Aznar’s centre-right-wing People’s Party government (1996–2004) [2,61]. Law 54/1997 on the Electricity Sector started the liberalization process of the electricity sector by opening networks to third parties, establishing an organized market for energy trading, and reducing state intervention policy in energy system management [2,52,61]. Spain has some positive figures in energy transition, even though its electricity prices are increasing, violating the EU and Spain’s goal of providing affordable energy prices for Spanish consumers [3,4,5].

However, the research and development (R&D) of Spain’s energy development heavily relies on top-down state planning and state funding, making the roles of bottom-up entrepreneurial initiatives missing. Spain’s National Energy and Climate Plan (NECP) and it is National RD&D Strategy (NRDD) have become Spain’s central energy R&D policy and guidance since 2007 and 2020 [4,73]. Based on EU energy principles, the NECP defines R&D, innovation, and competitiveness as one of its essential pillars. It also aims to enhance economic development and job creation through RE innovation [73]. The NRDD focuses on green hydrogen, energy storage, and RE innovation in mobility and industry [4]. However, as of 2018, Spain’s share of energy R&D spending is only 0.0085% of its GDP (the fourth-lowest share of GDP of all IEA member countries) [78]. While, as of 2017, private spending stood at 0.01% of GPD (slightly below the average value for the European countries with available data of 0.026%) [79]. Although Spain has put a massive effort into state R&D funding, it still does not have a higher performance than private R&D funding in the energy industry.

The Spanish central government plans to invest more state funding into energy’s R&D. The NECP’s two main projects, Research Challenges (Retos de Investigación) and Collaboration Challenges (Retos de Colaboración), accounted for EUR 154 million, almost 40% of R&D expenditure during the set period from 2020 to 2027 [2,73]. In other words, EUR 385 million in state funding would be injected into Spain’s energy R&D. The question is, due to the long and challenging economic recovery process in the post-COVID-19 era, whether the Spanish state can collect such a considerable amount of money for energy R&D (as economic recovery is the priority of the Spanish economic agenda). In addition, among the Research Challenges and Collaboration Challenges projects, RE received the largest share of expenditure, accounting for 63% of the total spending (50% of it was to wind energy, 26% for solar, 8% for ocean energy, and 6% biofuels) [2,73]. This implies that the Spanish state plans to subsidize the RE industry by taxation and other government expenditure methods. Yet, whether an industrial buddle would be created is another question that remains unanswered.

Industrial access restrictions might also impede Spain’s energy R&D. The Organization for Economic Cooperation and Development (OECD), the NRDD, and the NECP identify the exact problem [4,73,80]. First, the NRDD believes that Spanish enterprises and institutions have relatively low innovation capacity. Moreover, it also concerns the following issues that impede Spanish energy R&D: (1) the low public–private cooperation; (2) the low knowledge transfer between industry and society; (3) the lack of invention protections; and (4) the lack of using technology such as digitalization [4]. Further, the OECD and NECP identify the above phenomenon are caused by industrial barriers that the state policies conduct. 

The OECD Economic Survey considers the three following R&D-related problems that the Spanish entrepreneurs face [80]: (1) Different and complicated procedures to activate a public limited company (if opening an energy R&D firm is a difficulty, not to mention R&D as the further step). (2) Difficulty to open up and run the related retail business due to the restrictive licensing requirements (if selling becomes difficult, it would indirectly disincentivize R&D). (3) Entrepreneurship rates reveal potential entrepreneurs (those expecting to start a new venture in the next three years) are relatively scarce in Spain compared to the EU average (6.9% vs. 12.6%). This makes Spain’s energy R&D relatively insufficient compared to other countries. OECD stresses that further industrial openness to entrepreneurs is necessary. The NECP further proposed two leading policy suggestions to remove the industrial access restrictions that impede the energy R&D: (1) increasing the flexibility of hiring practices, including adapting them to the duration of innovation initiatives; and (2) streamlining the financial management of projects and initiatives through internal accounting.

### 4.2. The FIT-FIP Systems: A Mixed State Interventionist Energy Institutions

Spain started its legislation and liberalization process of RE and electricity sectors in the middle of the 1990s, which was almost in the same period as the UK (1989), Denmark (1996), and Germany (1998) [7]. Since 1997, Spain has passed legislation (Law 54/1997 [52]) to support RE development along with its liberalization process of the electricity market [2,61,74,81], while previous legislation (RD 2366/1994 [82]) only approved less than 50 MW of power generation with RE voluntarily to provide power to the autonomous communities for their approval [61]. Due to Law 54/1997 [52], producers of electricity from RE can access the energy network within the scope of this legislation, and the technical and economic conditions between producers and distributors are also defined and regulated [61]. In other words, despite a certain degree of liberalization, the Spanish state still set some industrial access restrictions. Like other liberalization policies of Premier Jose Maria Aznar’s government (1996–2004), the energy and electricity liberalizations are just a partial reform. Aznar’s reforms were incomplete compared to the privatization and marketization reforms carried out by US President Ronald Reagan and UK Prime Minister Margaret Thatcher, in the 1980s [83].

Spain’s energy system is based on a state subsidy scheme that has two essential components: feed-in tariffs (FIT, a state subsidy-taxation pattern) and feed-in premiums (FIP, a premium payment on top of the electricity market price; a maximum and minimum price control) [74]. The FIT-FIP system was introduced in Spain based on Law 54/1997 [52,61,82,84]. It was functioning until January 2012. During that period, operators of new renewable power plants had to choose between the FIT and the FIP: they were paid on top of the wholesale electricity price [37,74,85]. The scheme covered all major RE technologies except for solar photovoltaics (PV) (which was eligible for FITs only) [74].

Traditionally fixed-FIT systems are considered to have the following merits economically, providing long-time stability of RE investment [86]. (1) Investment security might be increased due to the FIT system. As the prices are fixed, it could create a certain degree of investment security by reducing the uncertainty the market prices’ fluctuation causes [37]. (2) Small and risk-averse investors might participate more in the FIT system. As uncertainty and risks are reduced, the predictability of future cash flows in the FIT system could be increased. The lower risk and uncertainty incentivize smaller and more risk-averse investors to participate in energy production, helping to facilitate RE project financing for non-traditional investors [37,62].

However, it has been argued for a long time that the FIT system could distort electricity prices and create economic burdens for electricity consumers. We consider that these arguments concur with free-market environmentalism and reflect the economic science’s fundamental principles of price functions. The first argument is that the fixed-FIT system distorts electricity prices [37,86]. Previous research considered that as the purchase prices offered under the FIT system remain fixed over time, the system could not reflect the price trends of the electricity market. Actual market prices would not be shown. If electricity prices decline dramatically, the RE producers will still receive the same guaranteed prices, leading to higher electricity prices for electricity consumers. If prices are fixed as state policies, it is not easy to modify them in the short run. As one study indicates, “once specific price paths (i.e., level, structure, and duration) are specified, changing those paths is both difficult and costly, as it creates excessive regulatory uncertainty that, in turn, increases investment costs” [86]. Therefore, the real and low electricity costs will not be received by electricity consumers, and the consumers must pay high and artificial electricity prices due to the FIT system.

The second argument concerns that fixed electricity prices under the FIT system overlook electricity demand [37]. Previous studies concerned with the fixed FIT prices’ ignorance of electricity demand concur with the FME approach and reflect the economic science’s fundamental principles of price functions. Economic science concerns prices as the signals of entrepreneurial productions [18,87,88]. The entrepreneurs produce and sell their products by speculating the prices their consumers might accept. In contrast, the consumers also decide if they prefer to buy the product or do not want to spend their money. If other conditions remain the same, the decreasing prices might create and increase demand for the product. However, as price signals are missing, electricity consumers cannot decide whether they want to buy electrical energy. The economic calculation becomes impossible.

The third argument is whether the politicians can make better judgments in selecting which energy enterprise or innovation is good for the energy transition in the long run [86]. This thesis involves the impossibility of centrally planned economic calculations as we have just discussed and related to the zero-sum game ending the theory of free-market environmentalism concerns. As shown in Section 2.1, zero-sum games are created through public policies and legislative decisions, where the market might have solved these problems. Governmental orders substitute voluntary contracts and actions [8,20,21,22]. Voluntary negotiations might solve conflicts. However, state legislation can cause the unexpected “one party wins, and the other loses” consequence. Moreover, incomprehensible legislation can cause the inefficiency of resource allocation through interventionism and regulation policies such as taxation, subsidies, and industry access restrictions. The result is that there is no way that consumers and producers can internalize the costs and benefits of environmental protection-related production, and a zero-sum game is created by state legislation. In the case of the energy transition, potential interest conflicts might be made between the selected energy enterprise A and the knocked-out energy enterprise B. Many energy consumers might not be satisfied with enterprise A, but their legislation has decided what type of RE the consumers should use. Artificial high price tags are economically wasteful and could “also raise political opposition to a well-intended policy” [86]. All the potential conflicts above could be avoided by the market choices of energy consumers and voluntary negotiations.

On the side of the FIP system, the traditional view is that it could help create a more harmonized electricity market, gradually and effectively removing the difference between renewable and conventional electricity [37,64]. Contrary to the FIT system, the FIP system has a more pro-market and flexible pricing mechanism. “Under the fixed-FIT option, the difference between the peak and off-peak tariff is small (amounting from 6.6 to 13.43 D/MWh) in comparison to the changes in the electricity market prices during peak and off-peak hours (average difference from 20 to 30 D/MWh)” [37]. The energy suppliers have less incentive under the FIT system as the electricity prices are not dynamically adjusted based on the market demand, giving them less incentive to provide more and better RE productions. On the side of the FIP system, electricity prices are decided and distributed hourly, reflecting the prices of peak and off-peak hours. The assumed cap and floor prices do not counteract the effect of the hourly determined electricity prices. The electricity demand could be reflected better in the FIP system than in the FIT. Thus, the FIP system provides a more pro-real market prices mechanism than the FIT system.

Seeing the results of reflecting better market prices, the Spanish government partly made a minor reform to liberalize the price decision mechanism. In 2007, the legislation RD (661/2007) set a cap and floor system for facilities under the premium option, which turned the premium into a variable payment [37,89]. The new system adjusts the premium hourly depending on the market price and the cap and floor values [37,89]. Although it is still an interventionist policy, the premium prices are reflected more than the accurate market prices.

Nevertheless, it is still argued that the FIP system has a deficiency, as the fluctuating prices might create a burden and uncertainty for energy consumers. Under the FIP system, the premium becomes smaller while the electricity prices increase, representing fewer burdens for consumers. However, while the prices decrease, the results would be the opposite. As the premium becomes bigger, electricity consumers must pay more. How to solve the fluctuation of prices remains an unsolved challenge for all the current FIT-FIP models. Section 5 will provide a free-market premium solution that might be a future path to reforming the current FIT-FIP systems.

However, the Spanish government abolished the FIT-FIP systems due to RD-Law 9/2013 [90]. In 2014, based on RD 413/2014 [91], both of the designs were replaced by a more market-oriented initiative on remuneration for investment and operation of the RE plants, especially for “Combined Heat and Power” [5]. The new system remains the feature of state subsidies. The new state subsidies aim to attain the minimum required to recover their investment and operating costs (including technologies with higher operating costs such as Solar Photovoltaic, Solar Thermal, and CHP [5]. In 2015, a further reform (based on Orden IET/1045/2014 [92]) aimed to use auctions to let energy enterprises enter RE markets. From the perspective of free-market environmentalism, although the new scheme since 2012–2013 has kept state subsidies, taxation, and industrial access restriction elements, the degree of the above interventionist policies has been reduced. This result is positive for Spain’s energy transition.

It should be pointed out that both the FIT and the FIP systems have market elements [37]. Under the two systems, the energy enterprises provide energy supply. Although the state intervenes in energy markets through taxation, subsidies, price regulations (which are executed through both taxation and state subsidies), and industrial access restrictions [7], they do not directly nationalize national resources or confiscate private properties as public goods such as Soviet socialism or communism [25,93,94]. Prices and the supply–demand market mechanisms still function to a certain degree. From the perspective of free-market environmentalism, removing FIT and FIP’s interventionist elements such as taxation, subsidies, price regulations, and industrial access would restore the market functions of energy prices, making energy transition economically more efficient. On the side of the 2013–2014 market reform under Mariano Rajoy’s center-right People’s Party government, electricity has been decided more by market elements. We believe that this is a further step toward an FME-based RE transition. However, high taxations, state subsidies, and state price regulations are the three main problems that impede Spain’s energy transition, causing relatively high electricity prices.

### 4.3. Electricity Prices, High Taxation Level, and Energy Cost in the Post-Pandemic Era

Although officially, Spain’s electricity tax component of its prices is relatively low among most of the IEA countries, which was 5% for industrial users and 21% for houseful users [2], the hidden tax has been a server component of why Spain’s electricity prices are relatively high among the listed 28 IEA countries. Spain has the eighth highest electricity prices for industrial uses (122.7 USD/MWh) and the fifth highest (287.7 USD/MWh) for household consumers in 2019.

Due to the Spanish Official State Gazette’s Order IET/843/2012 [46] and Resolution of June 28 of 2012 [47], it is calculated that as of 2015, although Spanish industrial users’ electricity tax component is only 3.87% of industrial users’ electricity prices, it does not include a 21% VAT a 22.8% taxation for RE subsidies, a 4.79% taxation for the cost of transporting the electricity to the Balearic Islands and the Canary Islands, and an around 7.87% taxation component of the bill that is to pay the tariff deficit for closing nuclear power plants [48]. As of the middle of 2012, Spain’s industrial users had to pay altogether around 58% taxation of the total electricity prices. Only around 40% of the electricity prices that industrial consumers paid were the real cost of electricity.

**Figure 5:**
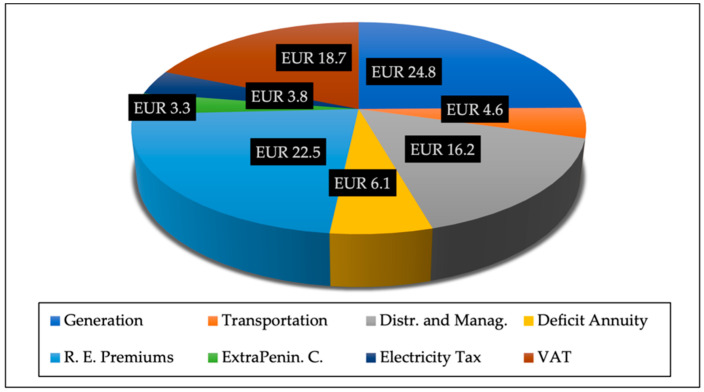
Spain’s electricity bill for industrial users as of the middle of 2021 (per EUR 100). Source: Own elaboration from CNMC [95].

**Figure 6:**
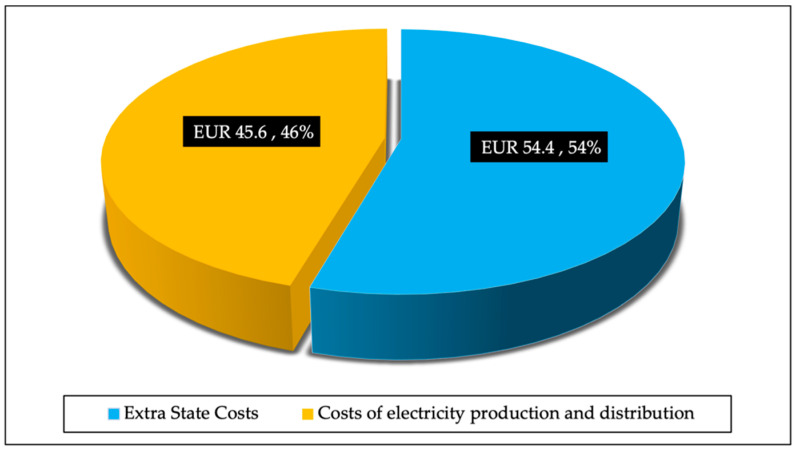
The percentage of the electricity bill corresponds to costs of production and distribution of electricity and extra costs of the Spanish State as of the middle of 2021. Source: Own elaboration from CNMC [95].

Our calculation shows similar patterns as the above references indicated. Figure 5 presents Spain’s electricity bill for industrial users as of 2021. Among a EUR 100 electricity bill, the real taxation components are EUR 54.4 (including VAT, electricity tax, premiums, extra peninsula transportation subsidies, and the tariff deficit for closing nuclear power plants). Only EUR 45.6 is the real electricity cost (including electricity generation, transportation, and distribution management). To make the real taxation vs. real cost scenario clear, Figure 6 shows the two proportions.

For household electricity prices, the IEA indicates that Spain is also among the highest in IEA countries (fifth overall), at 287.7 USD/MWh, as data of 2019 show, with (officially admitted) taxes accounting for 21% [2]. The same pattern has been maintained in the last few years. In September 2021, apart from the 13.5% regular taxation and the 51.5% energy cost, the Spanish citizens must pay 35% “tolls and charges” (peajes y cargos in Spanish) [49]. Tolls refer to the cost of transport and distribution networks, and the charges refer to the premiums for renewables, extra-peninsular subsidies, and compensation for the tariff deficit [50]. In other words, Spanish citizens pay 48.5% of total taxation for their energy consumption. As of March 2022, as other EU countries such as Denmark, Germany, and Portugal are reducing their electricity prices, Spain still maintains its electricity price levels [51]. In this regard, Spain keeps a high electricity taxation component compared with other developed countries.

### 4.4. State Subsidies

**Figure 7:**
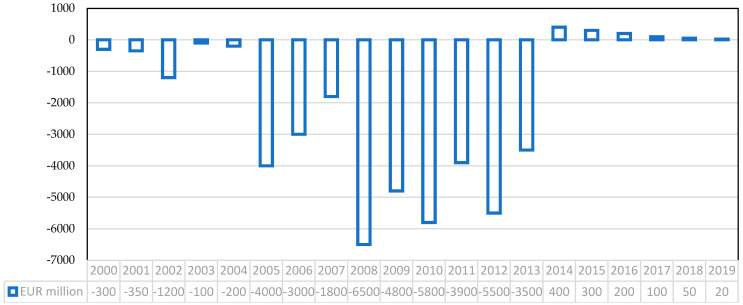
Spain’s electricity system tariff balances from 2000 to 2018. Source: Own elaboration from Spanish government response to the IEA questionnaire in [2].

Although in Section 4.2, we discussed Spain’s previous energy taxation-subsidy mechanism FIT-FIP systems, it is essential to perceive the general state subsidy institutional arrangements for energy and RE industries. Spain has decades of history of subsiding REs, which peaked at EUR 6.3 billion in 2008 [2]. However, as the FIT-FIP systems were abolished in 2012–13, Spain has entered a new era with fewer state subsidy programs than the previous FIT-FIP institutions. Since its energy reform in 2013, Spain’s electricity system has turned a loss into a profit, and a small surplus was reached in the 2014–2018 period [2]. Figure 7 below shows Spain’s electricity system tariff balances from 2000 to 2018. Data since 2019 are still not available from the IEA.

Despite abolishing the two systems in 2012–2013, the Spanish government still provides energy subsidies. To promote electrification, in December 2020, the Spanish government drafted a law establishing a national fund to subsidize the electric industry, its REs, cogeneration, and waste [2]. Before that period, the Spanish government directly subsidized local projects to promote a low-carbon economy through the European Regional Development Fund program, valued at EUR 3.28 million [2]. Subsidies for coal–nuclear closures continue (the coal subsidy ceased in 2018 due to the EU requirements), and one funding is through taxation on electricity consumption. Subsidies for oil products continue. As of 2019, it amounted to around EUR 1087 billion, “close to 50% of which went to the farming sector in the form of subsidies for fuel used for agricultural production while an additional one-fourth were tax exemptions for kerosene used in air transport” [2,96].

Like the FME approach, the IEA also does not recommend such subsidies. It argues that “the Spanish government should concentrate on improving flexibility in the market and creating proper price signals for investments” [2]. The Spanish state has verbose, redundant, and complicated regulatory documents compared with other developed countries, such as Germany, Denmark, and the UK [7]. This experience also concurs with the IEA’s suggestion that “it remains important for Spain to work on a clear and effective regulatory framework” [2]. The Spanish government should modernize and simplify its legislative documents, clarity, and accessibility. No country will succeed in reform with inefficient government work and outdated legal documents.

### 4.5. Industrial Access Restrictions

State industrial access restrictions have formalized another state-interventionist policy in Spain’s energy industry. As shown in Section 4.2, both the abolished FIT-FIP systems contained state industrial access restriction elements. However, general state industrial access restrictions still operate in Spain, which impede market competition and prevent energy transition.

The solar PV industry is one of the cases of industrial access restrictions. After the FIT payments were eliminated, pressure groups demanded that the government’s industrial policy be tilted toward solar PV [37]. Caps on the annual installed capacity for this technology were imposed, which was retroactive to the measures of eliminating previous FIT-FIP institutions. A previous study criticized that “The surge in PV projects put unexpected pressure on government coffers, and forced a drastic revision of the policy, which significantly increased the risk perception of Spain’s RE policy for investors and manufacturers” [37]. The new subsidy mechanism could also phase in other RE production, which was not under the consideration of the pressure groups, causing further state industrial access restrictions.

Table 4 shows the evolution of the market power of energy supplied for the domestic segment. It also clarifies the level of market concentration through the Herfindahl–Hirschman Index (HHI). A ratio above 2.500 indicates that an oligopolies electricity market is formed due to Spain’s state industrial access restrictions to the entrepreneurial competition in the energy industry (ratios between 1000 and 1800 points reflect a competitive marketplace, ratios between 1800 and 2500 for a concentrated marketplace (oligopoly). Above 2500, the market could be considered highly concentrated (monopoly). In 2019, “85% of energy production, 100% of the distribution network, and 90% of final sales are controlled by four giant firms” [32,39]. There four giant firms are: Endesa, Iberdrola, Naturgy, and EDP. Although the share of small energy production and trading companies has grown by 75% in the last decade, the four giants only reduced their market power by 10% (from 94% to 85%). In contrast, other small energy enterprises with better RE innovation are excluded from the Spanish energy market [32,63].

Although CNMA claimed that Spain’s energy and electricity is a “free market” [39], both the FME theory and the empirical evidence oppose this opinion. A market would never be free if the state imposes vital state industrial access restriction elements. As the FME approach indicates, this policy distorts market price coordination. Spanish energy enterprises must calculate what benefit they can receive from the state-interventionist policies instead of concentrating on how to serve their energy consumers better. Moreover, as economic theories show [18,87,88], an oligopoly market based on state interventionist policy impedes market competition and violates consumer sovereignty, causing uprising prices due to oligopoly–monopoly institutions. Fortunately, on 31 March 2022, Premier Pedro Sanchez’s left Spanish Socialist Working Party (PSOE) government decided to streamline permits for utility-scale solar, supporting another 7 GW under self-consumption [97]. This action is a positive sign for Spain to gradually eliminate state industrial access restrictions for energy and RE industries. We encourage the Spanish government to further executive the same path of policy.

## 5. Discussion: Reform Agenda, Research Limitation, and Future Research

### 5.1. Reform Agenda

In the above sections, we analyzed the general situation of Spanish energy transition and its increasing electricity prices in recent years. This section proposes a reform agenda for Spanish energy transition based on free-market environmentalism to reduce electricity costs and prices. In the first place, previous studies and our conclusion support the Spanish government’s phase-out of FIT-FIP systems. As we indicated in Section 4.2, the two systems have distorted electricity prices [37,86], overlooked electricity demand [37,65], damaged energy innovation [86], and created burden and uncertainty for energy consumers. A previous study even further indicated that such subsidies through FITs could even create welfare losses for society, “not only do such subsidies distort electric markets and reward inefficient [RE technologies] developers and operators; they negatively impact electricity consumers because they are a tax that increases as the overall share of [RE technologies] increases” [86]. In this regard, high energy and electricity prices caused by state-interventionist policies can also distort the competition of different industries due to the increasing energy costs (in the case of Spain, i.e., the tertiary sector and automobile industries). In addition, although FIT-FIP systems try to provide subsidies to eliminate market uncertainty (i.e., price inflation and interest risk [86,98,99,100], they can never eliminate market uncertainty as it is the nature of the market [16,88]. Even the relatively successful German FIT system was criticized for its adverse impact on electric rates and distortion of prices [7], as German retail consumers have increasingly protested against it [86]. The well-known American economist Frank Knight’s law on the uncertainty inherent in economic activities is also worthy of consideration and reference by energy policy scholars [101].

However, secondly, as market uncertainty always exists, from a free-market environmental perspective, it is still necessary to establish a market-based institution to hedge against the impact of market price fluctuations on the energy industry. Lesser and Su [86] proposed the following four measurements: (1) use proven market mechanisms to elicit truthful information, (2) ensure installation efficiency and generation efficiency both in the short term and in the long term, (3) guarantee the timely achievement of policy goals, and (4) be easy to implement and monitor.

Langniß et al. introduced three pro-market models that might be constrictive for the energy transition: the Retailer Model (retailers directly adopt RE supplies and interact with consumers); the Market Mediator Model (the mediator is a profit seeker, who is incentive inducted making economic efficiency of energy transition possible) through auctions; and the Optional Bonus Model (the model as a supplementary method is renewed every year or every month through energy suppliers’ preferred remuneration with a minimum price floor) [65]. Spain introduced the Optional Bonus Model between 2005 and 2006 [65]. However, Langniß et al. pointed out that this fixed price does not benefit the Spanish energy consumers as they must pay higher costs. Therefore, although the above scholars have tried to design a more pro-market FIT system that could enhance the energy industry’s competitiveness and reduce energy consumers’ costs, they all admitted these plans have shortcomings and could not entirely reflect the dynamic energy market, making planning impossible. Therefore, it is worth studying whether it is possible to hedge price fluctuations by establishing a capital pool or an insurance mechanism to form an utterly market-based price risk hedging mechanism that may help Spain’s energy transition and the entire market-oriented energy transition.

Thirdly, taxation, state subsidies, and state industrial access restrictions on energy and RE production should all be eliminated as much as possible. The hidden tax has been a component of why Spain’s electricity prices are relatively high among 28 IEA countries. Our research, along with the IEA paper [2], proposes that Spain adopt a market price mechanism as much as possible, considering the deficiencies of the previously ceased FIT-FIP systems, as they have distorted electricity prices. We recommend the Spanish government modernize and simplify its legislative documents for clarity and accessibility.

State subsidies on the energy industry should be ceased. The deficiencies of the previous FIT-FIP systems have distorted electricity prices [37,86], overlooked electricity demand [37,65], damaged energy innovation [86], and created burden and uncertainty for energy consumers. Although they were abolished in 2012–13, the Spanish government still provides energy subsidies for electrification, coal–nuclear closure, and oil products, as mentioned above. For state industrial access restrictions, although both FIT-FIP systems, as state industrial access restriction institutions, have been abolished since 2013, the general state industrial access restrictions still operate in Spain. A total of 85% of energy production, 100% of the distribution network, and 90% of final sales are controlled by four giant firms (Endesa, Iberdrola, Naturgy, and EDP) [32,39]. Although CNMA claimed that Spain’s energy and electricity is a “free market” [39], both the FME theory and the empirical evidence oppose this opinion. A market would never be free if the state imposes substantial state industrial access restrictions as it impedes market coemption preventing the energy transition. An oligopoly market based on state interventionist policy impedes market competition and violates consumer sovereignty, causing an uprising price due to oligopoly–monopoly institutions.

Fourthly, Spain should also enhance its research on energy transition based on market forces, as a previous OECD study indicated. The 2018 OECD report shows that only 3% of all capital invested in start-ups in Spain between 2011 and 2018 focused on digital and artificial intelligence technologies, far behind France (13%), Germany (14%), and the UK (55%) [67]. However, it is understandable that private funding would be squeezed out if the Spanish government devoted more state funding to energy research. The more the Spanish state uses taxation, the less money that its citizens and entrepreneurs can administrate for private-based RE research. As the FME approach indicates, the taxation and state subsidies on energy research breach private property rights and impede entrepreneurship. Therefore, it is crucial to gradually eliminate the state funding in RE research, thus creating an accessible environment for energy and environmental analysis based on entrepreneurship.

Fifthly, Spain should conduct further free-market reform to create better energy and RE entrepreneurial innovation environment. After its democratization in the 1980s, although Span made a partial market reform under the Jose Maria Aznar government from 1996 to 2004 [102], partially liberating its electricity market, Spain is still ranked lower in economic freedom than other developed countries. The Heritage Foundation’s 2022 economic freedom index shows that Spain’s economic freedom score is ranked 26th among 45 European countries. Its overall score is below the regional average but above the world average [103]. Among the data, Spain is ranked 32nd of 180 countries in Transparency International’s 2020 Corruption Perceptions Index, but its overall score (62) is one of Western Europe’s lower scores [103]. A more pro-private property and pro-market forces institutional arrangements would promote economic development and entrepreneurial innovation in any industry, and vice versa [92,93,104]. In short, we recommend Spain learn from the cases of Germany and Demark to provide spaces for the local community and firm levels initiatives and the UK’s experience of low electricity taxation to reduce energy costs and enhance a better and more innovative RE transition.

Sixthly, Spain should adopt proper methods to face the energy crisis caused by the 2022 Russian invasion of Ukraine. The previous price inflation caused by the post-pandemic monetary expansion, the Ukrainian War, the international sanctions on Russia, and other uncertain factors make it challenging to handle the current energy transition agenda [100,105]. Unfortunately, the Spanish government continues the previous interventionist energy policy. On 30 March 2022, the governments of Spain and Portugal presented a preliminary proposal to the European Commission that established a reference price for gas of EUR 30 per megawatt (MWh) to lower the price of electricity [106]. This price was four times lower than the actual prices during the week that the Spanish government made the proposal. Economic science indicates that this policy would result in a larger governmental deficit, and uncertainty might be created in the long run. However, as previous studies have shown, conventional backup sources such as natural gas, coal, or nuclear might also be used to reduce energy prices in the short run before achieving a 100% energy transition [2,31]. The EU has listed natural gas and nuclear energy as clean energy since February 2022 [56]. They could be alternative energy options for Spain instead of more interventionist policies. A policy that ignores people’s livelihoods for energy transition is questionable from a political economy perspective. A policy to achieve the short-run policy goal of reducing prices without considering its long-run damage to the energy market and energy transition is also doubtful. Table 5 compares our policy implications and the corresponding empirical findings.

### 5.2. Research Limitation and Potential Future Research

Although several policy suggestions have been provided above, we still consider that there are some limitations that our research does not cover. They should be studied in future research. In the first place, our current research does not cover the price inflation effects caused by the European Central Bank’s expansionary monetary policy after the outbreak of the COVID-19 pandemic. The FME approach’s policy effectiveness of providing affordable energy prices is under the condition of regular monetary policy when the central banking institution does not trigger expansionary monetary policy [107]. This would have three possible effects [108,109]. (1) General price inflation will happen due to the effects of expansionary monetary policy. Moreover, the productive structure would be distorted as newly injected money goes to the specific industries supported by the new financed money. These industries would not be invested under the unaggressive monetary policy. (2) The entrepreneurial calculation would also be negatively affected as the entrepreneurs might favor investing in the unprofitable economic sectors if there is no artificial injected money invested. Malinvestment is caused by expansionary monetary policy. (3) The financial markets would also be misled to invest in the sectors supported by expansionary monetary policy. Therefore, the financial orders would also be distorted. As we have seen in our paper, the current price inflation situation has already affected Spain’s energy and electricity prices. Thus, it is necessary to explicitly answer how the post-pandemic expansionary monetary policy affects the EU and the global energy sector.

Secondly, as we have mentioned in Section 5.1, the current Russian invasion of Ukraine has also triggered an energy crisis in Europe. As European governments are taking action to confront the war and sanction Russia, will government policies trigger more energy crises, and whether the FME approach is still applicable under such conditions should be resolved theoretically and empirically.

Thirdly, the FME approach should answer how its path applies to developing countries. As mentioned in the introduction, we consider that the FME approach is compatible with the developed economy, as they already have the economic foundation and motivations to pursue more environmentally friendly policy targets. However, as the Environmental Kuznets Curve shows [110], society sometimes starts to protect the environment after economic development has been achieved and where the environment has already been polluted. How does the FME approach apply to developing countries, especially their community [111] and bottom-up entrepreneurial initiatives [104]? The FME approach should resolve these questions.

## 6. Conclusions

This paper analyzed the general situation of the Spanish energy transition and its increasing electricity prices in recent years. Based on previous research on the EU’s free-market transition in the cases of Germany, Denmark, and the UK, this paper found that high taxes, high government subsidies, and government industrial access restrictions as being violations of private property rights that not only hinder the development of the RE industry in Spain, but also increase the cost of the energy and power industries, leading to Spain’s electricity prices remaining relatively high both before and after the outbreak of the COVID-19 pandemic. Our ARIMA model showed that Spain would fail the EU goal and Spanish *National Energy Poverty Strategy 2019–2024* to provide affordable energy prices as part of the green energy transition. We recommend that Spain learn from the cases of Germany to provide spaces for the local community and firm-level initiatives and the UK’s experience of low electricity taxation to reduce energy costs, enhancing a better and more innovative RE transition.

Evidence supports the Spanish government’s phase-out of FIT-FIP systems and previous studies. The two systems distorted electricity prices, overlooked electricity demand, damaged energy innovation, and created burden and uncertainty for energy consumers. As market uncertainty always exists, it is still necessary to establish a market-based institution to hedge against the impact of market price fluctuations on the energy industry from a free-market environmental perspective. It is worth studying whether it is possible to hedge price fluctuations by establishing a capital pool or an insurance mechanism to form an utterly market-based price risk hedging mechanism that may help Spain’s energy transition and the entire market-oriented energy transition.

This research found that taxation, state subsidies on energy and RE production, and state industrial access restrictions still impede Spanish energy transition and cause increasing energy prices. The hidden tax has been a component of why Spain’s electricity prices are relatively high among 28 IEA countries. Although the FIT-FIP systems were abolishment in 2012–2013, the Spanish government still provides energy subsidies for electrification, coal–nuclear closure, and oil products, impeding the price coordination and market competition of the energy and RE industries. For state industrial access restrictions, 85% of energy production, 100% of the distribution network, and 90% of final sales are controlled by four giant firms (Endesa, Iberdrola, Naturgy, and EDP). An oligopoly market based on state interventionist policy impedes market competition and violates consumer sovereignty, causing an uprising price due to oligopoly–monopoly institutions. Fortunately, on 31 March 2022, the Spanish government decided to streamline permits for utility-scale solar. This is a positive sign for Spain to gradually eliminate state industrial access restrictions for energy and RE industries. We encourage the Spanish government to further executive the same path of policy.

We also recommend that Spain should also enhance its research on energy transition based on market forces. Between 2011 and 2018, only 3% of all capital invested in energy start-ups in Spain focused on digital and artificial intelligence technologies, far behind France (13%), Germany (14%), and the UK (55%). However, it is understandable that private funding would be squeezed out if the Spanish government devotes more state funding to energy research. The more the Spanish state uses taxation, the less money that its citizens and entrepreneurs can administrate for private-based RE research. As the FME approach indicates, taxation and state subsidies on energy research violate private property rights and impede entrepreneurship as the driving force of the market economy. It is essential to gradually eliminate state funding in RE research, thus creating a free environment for energy and environmental analysis based on entrepreneurship. Finally, a policy that ignores people’s livelihoods for the sake of energy transition is questionable from the perspective of the political economy, while an approach to achieve the short-run policy goal of reducing prices without considering its long-run damage to the energy market and energy transition is also doubtful.

## Figures and Tables

**Table 3:** The ARIMA Model.

Model	ARIMA (1,0,1) with Non-Zero Mean		
Coefficients:			
	AR1	MA1	Mean
	0.7455	0.7414	101.533
s.e.	0.2927	0.2186	55.8519
Sigma’2 = 1347:	Log	Likelihood	−64.82

Source: Own elaboration from Statista and Rstudio [77].

**Table 4:** Evolution of the Spanish market power of energy supplied for the domestic segment.

Years	Endesa	Iberdrola	Naturgy	EDP	Viesgo	Repsol	HHI
2011	42%	35%	15%	2%	2%	0%	3.237
2012	41%	35%	16%	3%	2%	0%	3.173
2013	41%	34%	16%	3%	2%	0%	3.071
2014	39%	33%	17%	3%	2%	0%	2.943
2015	39%	33%	17%	3%	2%	0%	2.903
2016	38%	32%	17%	3%	2%	0%	2.796
2017	37%	32%	17%	3%	2%	0%	2.694
2018	37%	32%	15%	3%	2%	0%	2.609
2019	36%	32%	13%	4%	0%	3%	2.500

Sources: Adapted from [39].

**Table 5:** The comparison between policy implications and the corresponding empirical findings.

Policy Implications	Corresponding Findings
Previous studies and our conclusion support the Spanish government’s phase-out of FIT-FIP systems.	The two systems have distorted electricity prices [37,86], overlooked electricity demand [37,65], damaged energy innovation [86], and created burden and uncertainty for energy consumers.
As market uncertainty always exists, from a free-market environmental perspective, it is still necessary to establish a market-based institution to hedge against the impact of market price fluctuations on the energy industry.	Lesser and Su [86] proposed the following four measurements for confronting price fluctuations: (1) use proven market mechanisms to elicit truthful information, (2) ensure installation efficiency and generation efficiency both in the short term and in the long term, (3) guarantee the timely achievement of policy goals, and (4) be easy to implement and monitor. Langniß et al. [65] introduced three pro-market models that might be constrictive for the energy transition: Retailer Model, Bonus Model, and Optional Bonus Model.
Taxation, state subsidies on energy and RE production, and state industrial access restrictions should all be eliminated as much as possible.	The hidden tax has been a component of why Spain’s electricity prices are relatively high among 28 IEA countries. As of 2022, Spain’s industrial and household electricity users must pay around 58% and 48.5% of total taxation, respectively. The Spanish government still provides energy subsidies for electrification, coal–nuclear closure, and oil products [2]. 85% of energy production, 100% of the distribution network, and 90% of final sales are controlled by four giant firms due to industrial access restrictions [32,39].
Spain should also enhance its research on energy transition based on market forces, as a previous OECD study indicated.	The 2018 OECD report shows that only 3% of all capital invested in energy start-ups in Spain between 2011 and 2018 focused on digital and artificial intelligence technologies, far behind France (13%), Germany (14%), and the UK (55%) [67].
Spain should conduct further free-market reform to create better energy and RE entrepreneurial innovation environment	Although the Spanish made a partial market reform under the Jose Maria Aznar government from 1996 to 2004 [102], partially liberating its electricity market, it is still ranked lower in economic freedom than other developed countries. Spain’s economic freedom score is ranked 26th among 45 countries in Europe. Its overall score is below the regional average but above the world average [103]. Spain is ranked 32nd of 180 countries in Transparency International’s 2020 Corruption Perceptions Index, but its overall score (62) is one of Western Europe’s lower scores [103].
Spain should adopt proper methods to face the energy crisis caused by the 2022 Russian invasion of Ukraine.	The previous price inflation caused by the post-pandemic monetary expansion, the Ukrainian War, the international sanctions on Russia, and other uncertain factors make it challenging to handle the current energy transition agenda [100,105]. Unfortunately, the Spanish government continues the previous interventionist energy policy. It presented a preliminary proposal to the European Commission that established a reference price for gas of EUR 30 per megawatt (MWh) to lower the cost of electricity [106].

## Data Availability

Not applicable.
